# Sustainable H_2_‑Rich Syngas Production via Microwave-Assisted vs Conventional Catalysis of Pinewood

**DOI:** 10.1021/acs.iecr.5c02616

**Published:** 2026-01-05

**Authors:** Kshitij Tewari, Devin Burton, Brandon Robinson, Changle Jiang, Debangsu Bhattacharyya, Jianli Hu

**Affiliations:** † Department of Chemical and Biomedical Engineering, 5631 West Virginia University, Morgantown, West Virginia 26505, United States; ‡ Department of Physics and Astronomy, West Virginia University, Morgantown, West Virginia 26505, United States

## Abstract

Catalytic gasification of biomass is a promising method for producing hydrogen-rich syngas, which is a valuable resource for clean-energy applications. In this study, microwave-assisted biomass gasification was compared with conventional thermally driven biomass gasification using pinewood as the biomass without the use of external gasifying agents (such as air, steam, and CO_2_), under non-catalytic and catalytic conditions. The catalysts consisted of either an iron or a nickel catalyst, and the pinewood used as biomass contained 42% oxygen. This comparative analysis explores the differences in reaction chemistry, product yields, and the role of key reactions such as the water gas shift (WGS) reaction, Boudouard reaction, etc. The gas-phase and liquid-phase products were analyzed using online gas chromatography, and the fresh and spent catalysts were analyzed using X-ray diffraction (XRD) techniques. It was found that microwave-assisted gasification offers advantages in terms of enhanced reaction efficiency, catalyst stability, and hydrogen yield. For the microwave-assisted reaction, the gas yield reached 87%, the char yield was 12.1%, and the tar yield was less than 1% (0.793%) at 550 °C. In contrast, thermal-assisted gasification using the same catalyst produced a gas yield of 85.796%, char yield of 11.438%, and higher tar yield of 2.8% at 900 °C. The higher microwave-assisted performance was attributed to faster heating and better control over reaction conditions, higher reaction rates, and more favorable conditions for hydrogen production.

## Graphical abstract

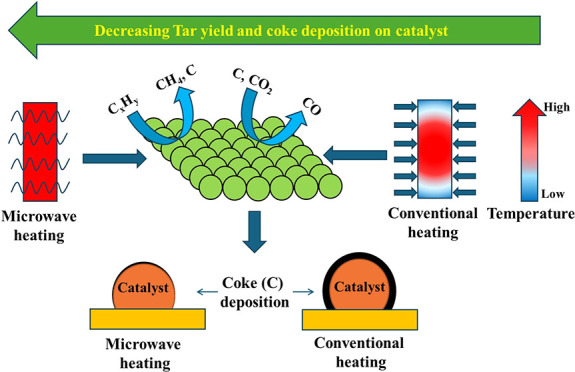

## 1 Introduction

Biomass gasification has emerged as a vital technology for transforming organic waste into useful energy products, especially hydrogen-rich syngas. In response to the growing concern about climate change and the increasing demand for renewable energy sources, this renewable energy source holds the potential to significantly replace traditional fossil fuels, providing a versatile energy carrier that can be utilized in various applications, including fuel cells, transportation, and chemical synthesis. The conversion of biomass into syngas represents a pathway toward low-carbon economy, aligning with global efforts to reduce greenhouse gas emissions and promote renewable energy usage. Biomass gasification is an innovative method that converts organic resources into syngas, a mixture of hydrogen and carbon monoxide, suitable as a valuable intermediate for fuel synthesis and chemical manufacturing.^1^

Hydrogen, as a clean fuel, yields only water upon combustion, making it an environmentally friendly alternative. When produced from biomass, it further contributes to carbon neutrality, as it utilizes carbon absorbed by plants during their growth process. Additionally, hydrogen-rich syngas can serve as a precursor for producing chemicals and fuels, making it a valuable component in the quest for sustainable energy solutions.^2,3^

Despite its promise, conventional thermal gasification encounters numerous challenges that can hinder its effectiveness. Thermal gasification, involving the conversion of biomass into syngas at high temperatures (typically above 700 °C), can suffer from several limitations, including low efficiency, prolonged reaction times, and the formation of tar and other undesirable byproducts. Tar, a viscous organic liquid formed during the gasification of biomass, can cause significant operational problems in gasification systems, including pipeline blockages, catalyst deactivation, and an overall reduction in syngas yield and quality.^4,5^

Therefore, exploring alternative gasification technologies is essential to enhancing syngas production. Microwave-assisted gasification is one of the most innovative approaches being investigated. Microwave heating provides volumetric, rapid heating at the reactor scale; however, at the microscale, dielectric inhomogeneity can generate local hot spots that accelerate bond cleavage. This method not only improves conversion rates and product yields but also significantly reduces the energy consumption associated with gasification.^6^

Microwave-assisted gasification presents several advantages over traditional methods. One of the key benefits is the ability to interact with the moisture content present in the biomass directly. Since moisture often impedes the gasification process, its effective management can enhance the efficiency of the overall conversion process. Furthermore, the microwave heating mechanism may assist in reducing tar production, thus eliminating additional processing steps required in conventional thermal gasification.^6–12^

However, microwave-assisted gasification is not without its challenges. The uneven temperature distribution caused by the nature of microwave radiation can lead to localized overheating or underheating, which can adversely affect the gasification process. Additionally, maintaining precise control of reaction parameters such as temperature and residence time is crucial to optimize the performance. The complexity of these processes highlights the need for thorough understanding and optimization of the microwave-assisted gasification pathway to fully harness its potential.^6,13^

The role of catalysts in the gasification process further complicates the comparison between microwave and thermal methods.^6,14^ Catalysts, which accelerate chemical reactions without being consumed, are pivotal to enhancing the gasification efficiency. They can lower the required activation energy for the reactions, enabling the gasification process to proceed at lower temperatures and reducing the formation of unwanted byproducts like tar. The implementation of catalysts not only improves the yield of hydrogen in syngas production but also allows for better control of the gas composition, promoting the generation of valuable products.^6,15^ Recent studies have used several catalysts for biomass pyrolysis or gasification, particularly nickel,^16–19^ iron,^20–22^ cobalt,^23^ molybdenum,^24,25^ and cerium oxide,^26,27^ and various supports^28,29^ for these catalysts, which are shown in Table 2. Hu et al. investigated the use of an activated carbon-supported Fe–Ni catalyst for the catalytic gasification of pinewood (PW) to produce syngas. They found that Fe–Ni/AC significantly increased hydrogen (H_2_) production and reduced carbon monoxide (CO) and methane (CH_4_) emissions, improving the H_2_/CO ratio. Catalytic gasification at lower temperatures (700–900 °C) enhanced gas composition control, and under optimal conditions (750 °C and 1 mL/min steam), the H_2_/CO ratio reached 1.97, the catalyst’s effectiveness varying depending on the difficulty in biomass feedstock decomposition.^30^ Thermal cracking of tar at 500–600 °C mainly produced light liquids, including water, while higher temperatures (650–700 °C) led to intense gas formation. Charcoal promoted cracking at lower temperatures, but its catalytic effect diminished at higher temperatures due to the intensified thermal effect. GC–MS analysis revealed that the primary liquid product consisted of oxygenated compounds from mild wood degradation. Charcoal decomposed these compounds at 500–600 °C. At higher temperatures, the steam gasified charcoal, increased the gas yield, and reduced the liquid output. Among various carbon samples, lignite-char (L-char) exhibited the highest catalytic activity for tar cracking and gasification compared to graphite and other chars.^31^ Recent studies have reinforced the importance of microwave-assisted catalytic gasification for sustainable hydrogen production. Cao et al.^32^ demonstrated that microwave heating enhanced energy efficiency while suppressing tar, whereas Erdemir et al.^33^ emphasized catalyst and reactor design strategies to maximize hydrogen selectivity under microwave fields. Catalyst development has also advanced, showing promoter-enhanced Ni activity in microwave dry reforming and improving reactor coupling, further guiding support selection for microwave-driven systems. Beyond catalyst and material innovations, process intensification through sorption-enhanced gasification with CaO sorbents has been reported to significantly improve the hydrogen yield.^34^ In parallel, Ni–Fe systems continue to attract attention for tar reforming under both microwave and thermal heating modes.

Despite the extensive research on biomass gasification, several important gaps remain in the existing literature. Previous studies have largely examined conventional thermal gasification and microwave-assisted gasification separately, and a direct, mechanistic comparison of microwave-driven and thermal catalytic gasification of pinewood under oxygen-limited conditions, without any external gasifying agents (air, steam, or CO_2_), has not been reported. In addition, the temperature-resolved evolution of syngas composition and the associated reaction pathways over Fe- and Ni-based catalysts under microwave heating remain insufficiently understood, as most prior studies focused primarily on final product yields rather than reaction chemistry. Furthermore, systematic evaluations of tar–gas conversion behavior and energy recovery efficiency under oxygen-limited microwave catalytic gasification and their comparison with conventional heating are lacking.

In this work, we conducted a detailed comparison of microwave-driven and thermal gasification of pinewood without external gasifying agents. Pinewood was used as the biomass feedstock, and it contained 46.66 wt % oxygen based on ultimate analysis (Table 1), corresponding to approximately 42% oxygen available for combustion and partial oxidation reactions; herein, oxygen acted as a limiting reactant. This study compared two heating methods: microwave heating and conventional heating. Under both heating modes, catalytic gasification of pinewood was carried out using Fe and Ni catalysts to investigate their effects on syngas composition, hydrogen yield, reaction kinetics, and energy recovery efficiency. This work therefore addresses the identified gaps and provides a framework for understanding catalytic pinewood gasification under oxygen-limited microwave and thermal heating conditions.

**Table 1:** Pinewood Biomass Elemental Composition (Obtained from the National Research Center for Coal and Energy, Certified for Standard Testing, West Virginia University)

ultimate analysis	
element	wt %
C	48.478
H	5.546
N	0.312
S	0
O	45.664

proximate analysis	
parameter	wt %
moisture	7.47
volatile	74.4
fixed carbon	18.075
ash	0.055

## 2 Materials and Methods

### 2.1 Materials

Pinewood was purchased from Lowe’s. This study used analytical grade chemicals. Nickel­(II) nitrate hexahydrate (99%) was purchased from Acros Organics, and ferric nitrate nonahydrate (98.6%) from Fisher Scientific. All of the reagents were used as received without further purification. All solutions of these chemicals were prepared using deionized water as a solvent. Tedlar gas bags (1 L, Thermo Scientific) were utilized for gas sample injections into the micro-gas chromatograph (Micro-GC, Inficon Inc.).

### 2.2 Catalyst Preparation

Nickel­(II) nitrate hexahydrate and ferric nitrate nonahydrate were dissolved in 100 mL of deionized water separately at room temperature with continuous stirring at 350 rpm. Both solutions were dried at 120 °C for 6 h, followed by calcination in the presence of nitrogen gas to convert metal nitrates to oxides and reduction in the presence of hydrogen gas to convert metal oxide into metal. For calcination, samples were packed in a 0.5 in. quartz tube with glass wool, heated at 10 °C/min to 400 °C under N_2_ flow (50 sccm/min) and held for 2 h. This process converted ferric nitrate and nickel nitrate to iron oxide and nickel oxide, respectively. For reduction, the temperature was increased to 650 °C for iron oxide and 450 °C for nickel oxide at 10 °C/min under H_2_ flow (50 sccm/min) and held for 2 h. This process converted the oxides to their respective metals.

### 2.3 Reactor Design

Two different batch reactor designs were employed for biomass gasification in this work: microwave-driven heating and conventional heating.

Microwave-driven lignocellulose pinewood gasification was carried out in a quartz tube reactor, which was positioned within the Sairem microwave system (Figure 1). The reactions were conducted at a frequency of 2.45 GHz, with a maximum power output of 900 W. The output power used during the reaction was 0.03–0.05 kW. Temperature measurements were taken using an infrared pyrometer, while the forward power was regulated by a proportional integral derivative (PID) controller. The microwave power and tuning were controlled via a Eurotherm controller. The catalyst and pinewood were mixed in a quartz tube according to a predefined ratio for pinewood gasification. The quartz tube reactor had an internal diameter of 2.54 cm, an outer diameter of 2.34 cm, and a length of 15.00 cm, as shown in Figure 1. In this experimental setup, the biomass was carefully processed through grinding and screening to achieve a mean particle size of 200 μm, with particles ranging between 150 and 250 μm. This particle size was selected to ensure consistency with those used in prior studies of fixed-bed reactors on smaller scales. Typically, 1.0 g of lignocellulosic pinewood and 1.0 g of catalyst were used. Gasification was carried out under ambient pressure conditions, specifically at 1.01 bar or atmospheric pressure. The catalyst loading of 1.0 g was not selected as an optimized value but was fixed based on the reactor volume, microwave penetration depth, and a fixed pinewood mass (1.0 g), in order to ensure uniform mixing, stable heating, and direct comparability between Fe and Ni catalysts under both microwave-assisted and conventional heating conditions. In the microwave-driven system, a minimum catalyst mass of 1.0 g was required to achieve effective microwave coupling and complete gasification in the designed reactor. Catalyst loadings below 1.0 g resulted in reduced gas yields and increased tar formation due to insufficient microwave absorption, whereas increasing the catalyst mass beyond 1.0 g did not lead to measurable changes in gas composition or conversion. Therefore, 1.0 g represents the minimum effective catalyst loading for the present microwave reactor configuration. Catalyst mass optimization was beyond the scope of this study and will be addressed in future work. The entire reaction setup, including the quartz tube, was purged with nitrogen gas at a flow rate of 100 mL/min for 30 min to ensure an oxygen-free environment. Nitrogen (flow rate: 5 mL/min) was used as the carrier gas. The system was checked for leaks before each reaction. Once the microwave generator was started, the temperature was gradually increased to 525 °C according to the specified ramp rates. The gaseous products were collected in a Tedlar bag and analyzed using a four-channel Micro-GC.

**Figure 1:**
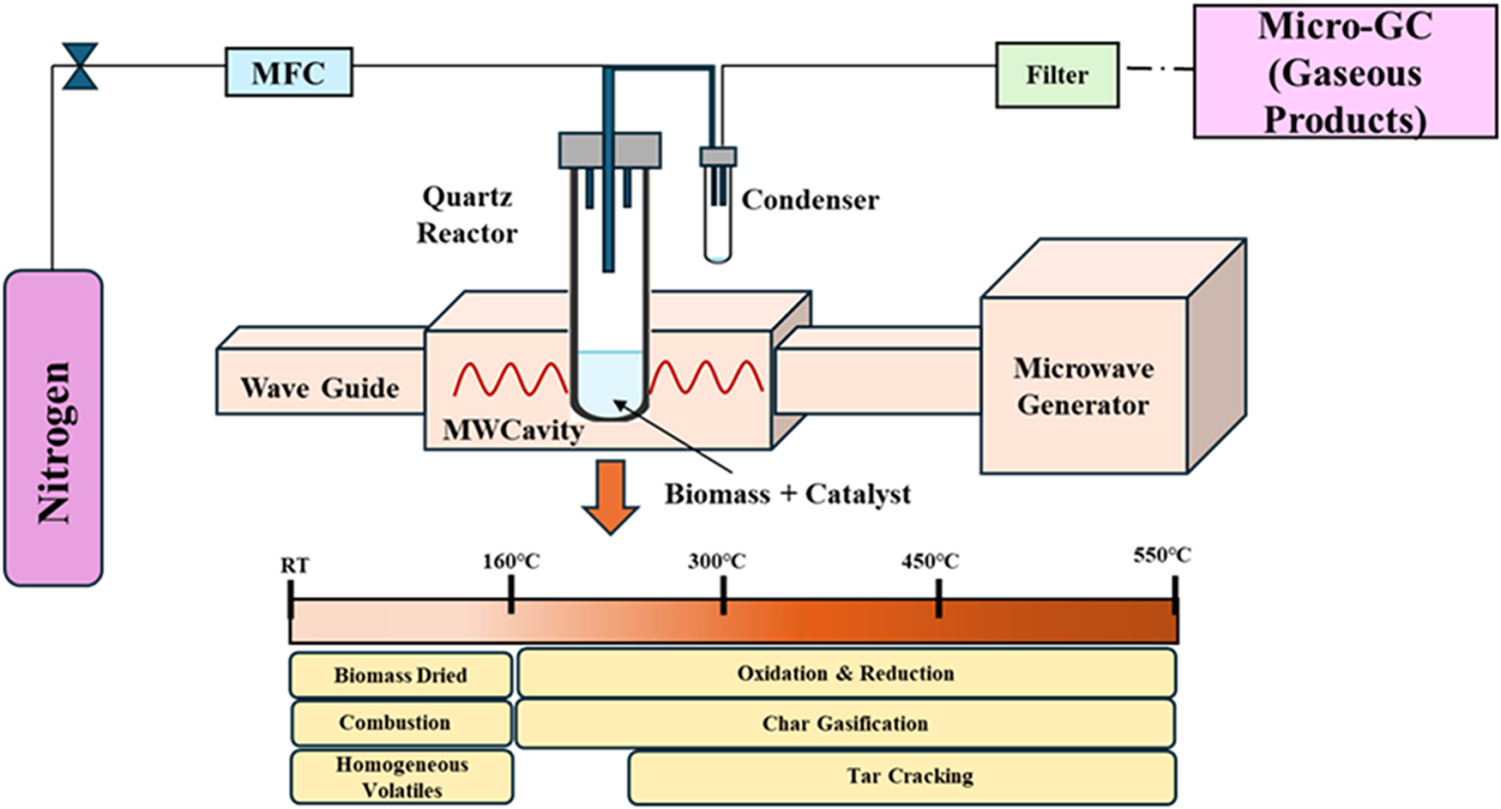
Schematic of pinewood gasification using microwave heating.

Gasification of pinewood via conventional heating was carried out in a fixed-bed reactor constructed from a 316 stainless-steel tube (Charleston Valve and Fitting). The reactor had an internal diameter of 10.5 mm, an outer diameter of 12.7 mm, and a length of 381.0 mm, as shown in Figure 2. For each experimental run, the amounts of pinewood and the catalyst were fixed to match the microwave experiments for direct comparison. The system was tested under various parameter configurations, with factors such as temperature, absence of an external gasifying agent, and the feed-to-catalyst ratio. The experimental procedure involved performing high-temperature reactions in a Carbolite furnace (USA), equipped with a programmable controller. A K-type thermocouple (Omega) was embedded in the reactor bed to monitor temperature, with the readings controlled through a PID controller, as depicted in Figure 2. During the reaction, the gas sample was collected in an SKC Tedlar 1 L sample bag and analyzed using a Micro-GC.

**Figure 2:**
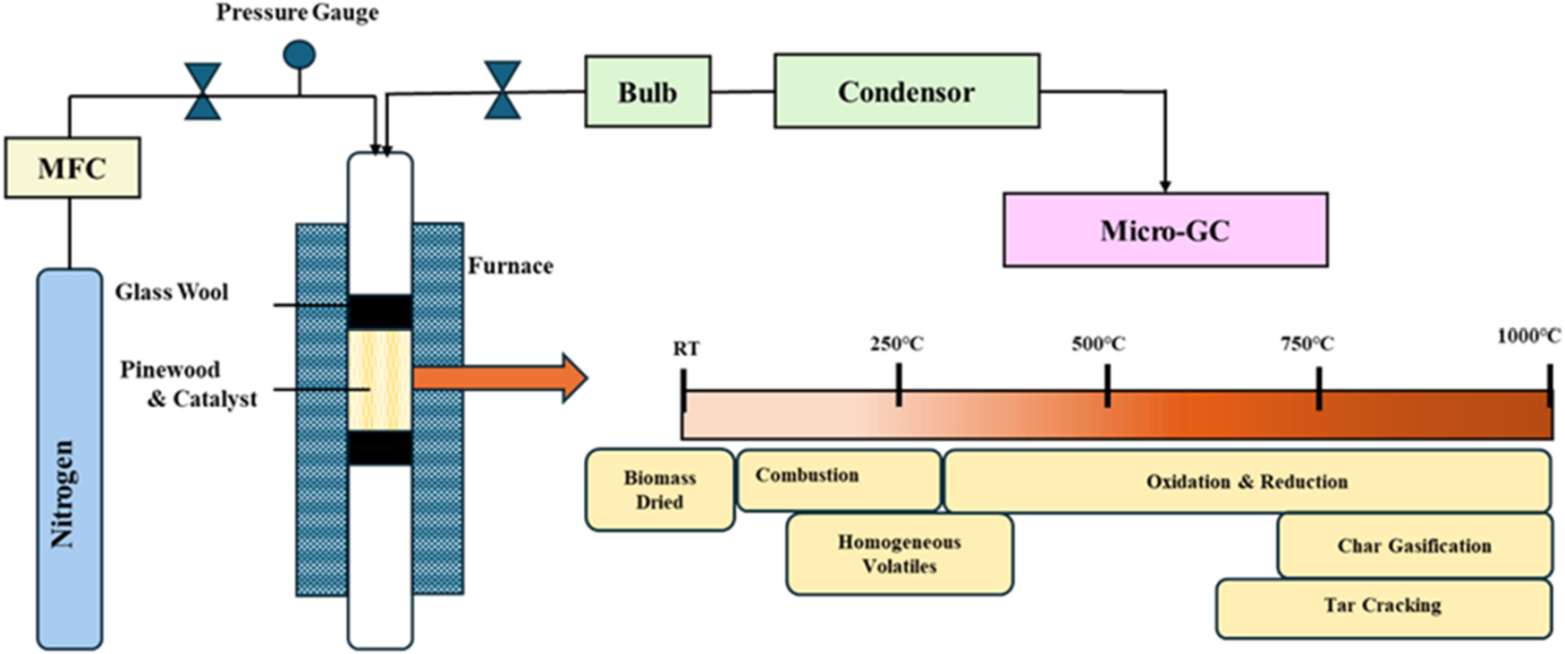
Schematic of pinewood gasification using conventional heating.

### 2.4 Elemental Analysis of Feedstock

The ultimate and proximate analyses of lignocellulosic pinewood are summarized in Table 1. Proximate analysis is essential for evaluating the biomass composition, including key parameters such as moisture content, volatile matter, fixed carbon, and ash. Moisture content is a critical factor in biomass handling, as it influences both the transportation efficiency and effectiveness of conversion processes. Volatile matter plays a significant role in thermal conversion, as it generates a higher volume of combustible gases. However, the presence of ash, inorganic components of biomass, can lead to operational challenges, such as fouling, agglomeration, and slagging in reactors, furnaces, and associated equipment.

In contrast, ultimate analysis focuses on determining the elemental composition of biomass, specifically carbon (C), hydrogen (H), nitrogen (N), and sulfur (S) content. The oxygen (O) content is derived indirectly by subtracting the sum of other elements from 100%. This in-depth analysis provides a thorough understanding of the fundamental characteristics of biomass, aiding in the optimization of its use and processing in energy-conversion systems.

Also, the higher heating value (HHV) is the total amount of heat released when a fuel is completely burned and the combustion products (including water vapor) are condensed back to liquid water. The Dulong formula is a commonly used empirical equation to estimate the HHV based on the elemental composition of biomass.^35,36^ The Dulong formula for HHV is

HHV=0.338×C+1.42×H+0.093×O

where C = weight % of carbon; H = weight % of hydrogen; O = weight % of oxygen (since nitrogen and sulfur do not contribute significantly to HHV).

Therefore, the higher heating value (HHV) is approximately 28.50 MJ/kg.

The lower heating value (LHV) is the amount of heat released during combustion, excluding the heat used to vaporize the water in the combustion products.^35,36^ To calculate the LHV, we use the following relationship

LHV=HHV−2.44×HHH

where HHH = weight % of hydrogen in the biomass.

Thus, the lower heating value (LHV) is approximately 14.96 MJ/kg.

Energy recovery efficiency describes the efficacy of a system in transforming waste energy into usable energy. It is defined as the ratio of energy recovered from a process to the total waste energy available for recovery.^37,38^

The lower heating values (LHVs) of syngas components at the STP are essential. Hydrogen (H_2_) and carbon monoxide (CO) possess LHVs of 10.1 MJ/m^3^. Methane (CH_4_), a vital component of syngas, possesses a higher LHV of 35.8 MJ/m^3^, providing it with a more energy-dense fuel. Conversely, carbon dioxide (CO_2_) has no energy content, as shown by its LHV of 0 MJ/m^3^, and it does not enhance the energy output of the syngas. For batch reactors, energy recovery efficiency is 35–50%. For this study, it is shown in Figure S1.^38^

### 2.5 Characterization

The *d*-spacing and crystal phases of the reduced and spent catalysts were analyzed by using X-ray diffraction (XRD). XRD data were acquired with a PANalytical X’Pert Pro X-ray diffractometer (PW3040), operating at 40 kV and 20 mA. The scan was performed over a range of 5–110° with a step size of 0.0167° and a scan rate of 5°/min.

## 3 Results and Discussion

### 3.1 Gasification Mechanism in Conventional Heating and Microwave Heating

Biomass gasification converts solid pinewood into valuable syngas that can be used for energy production or as a chemical feedstock. Syngas is a mixture of two major gases, hydrogen (H_2_) and carbon monoxide (CO). The remaining gases present were carbon dioxide (CO_2_) and methane (CH_4_). Biomass gasification has three main products, gas, char, and tar, as shown in eq 1. Both thermal and microwave gasification follow a similar sequence of 4 stages: (a) drying: removal of moisture from the biomass; (b) devolatilization: pyrolysis of biomass, leading to the release of volatile gases, including mainly water vapor, CO, CO_2_, CH_4_, and organic compounds, and leaving behind solid char; (c) gasification in which the char reacts with oxygen, steam, or other gases to produce syngas; (d) tar conversion: high-temperature reactions degrade complex organic compounds (tars) into lighter, more useful gases.

1 biomass→gas+char+tar

The difference between conventional and microwave gasification lies mainly in how heat is transferred to the biomass. Conventional gasification^14^ uses external heat sources such as combustion or electrical heating to raise the reaction temperature through a convective or conductive heat-transfer mechanism, while microwave gasification^39^ uses electromagnetic microwave radiation to directly heat the biomass and catalyst, causing biomass to rapidly decompose.

In thermally assisted heating, biomass is heated initially. Moisture is removed until 120 °C. Thereafter, volatile components are released (eqs 2–6) at temperatures up to 350 °C. Also, combustion reactions (eqs 7and [Disp-formula eq8]) take place simultaneously, which are highly exothermic. The remaining solid after this point is known as char. Heat breaks down cellulose, hemicellulose, and lignin into lighter compounds. This process occurs at temperatures between 300 and 500 °C. Cellulose, a polysaccharide, decomposes into levoglucosan (a sugar derivative) and other smaller organic molecules. Hemicellulose breaks down into aldehydes, acids, and furans, which are further decomposed into gases like CO_2_ and CO. Lignin, the complex aromatic polymer in pinewood, decomposes into a mixture of volatile organic compounds (VOCs), including aromatic hydrocarbons, phenolics, and tars.

2 $$s\mathrm{team reforming of methane},CH_{4}+H_{2}O\to CO+3H_{2}$$

3 $$w\mathrm{ater gas shift reaction},CO+H_{2}O\to CO_{2}+H_{2}$$

4 $$p\mathrm{artial oxidation},CO+0.5O_{2}\to CO_{2}$$

5 $$o\mathrm{xidation},CH_{4}+2O_{2}\to CO_{2}+2H_{2}O$$

6 $$p\mathrm{artial oxidation},H_{2}+0.5O_{2}\to H_{2}O$$

7 $$o\mathrm{xidation reaction},C+O_{2}\to CO_{2}$$

8 $$p\mathrm{artial oxidation reaction},C+0.5O_{2}\to \mathrm{CO}$$

From 500 to 900 °C, several chemical reactions take place, including pyrolysis and oxidation, reduction, and gasification (eqs 9–11), which produce syngas and other byproducts. These include hydrogenation, water gas reaction, and reverse Boudouard reaction, often requiring external heat or internal combustion of biomass.

9 $$h\mathrm{ydrogenation reaction},C+2H_{2}\to CH_{4}$$

10 $$r\mathrm{everse Boudouard reaction},C+CO_{2}\to 2\mathrm{CO}$$

11 $$h\mathrm{eterogeneous}\;w\mathrm{ater gas reaction},C+H_{2}O\to CO+H_{2}$$

12 $$s\mathrm{team reforming},C_{x}H_{y}+xH_{2}O\to x\mathrm{CO}+\left(0.5y+x\right)H_{2}$$

13 $$p\mathrm{artial oxidation},C_{x}H_{y}+0.5xO_{2}\to x\mathrm{CO}+0.5yH_{2}$$

14 $$\;d\mathrm{ry}\;r\mathrm{eforming},C_{x}H_{y}+CO_{2}\to 2x\mathrm{CO}+\left(0.5y+x\right)H_{2}$$

15 $$m\mathrm{ethanation},C_{x}H_{y}+H_{2}\to xCH_{4}$$

16 $$c\mathrm{racking},C_{x}H_{y}\to 0.25y{\mathrm{CH}}_{4}+\left(x-0.25y\right)C$$

Tar composition changes with temperature, starting with mixed oxygenates and evolving into more complex polyaromatic hydrocarbons. Additionally, the duration of gasification affects tar conversion into syngas (eqs 12–16), with longer exposure leading to more cracking and production of gases and low-molecular-weight compounds.^40^ Char, a byproduct of biomass gasification, undergoes changes in its structure and carbon content with temperature, becoming more carbon-rich and aromatic as the temperature rises. Overall, gasification is a complex process that enables biomass to be used for sustainable energy production and industrial applications.

The microwave gasification process offers several advantages over conventional thermally heated gasification, including faster heating rates, more efficient energy transfer, and potentially lower reaction temperatures. Microwave heating is initiated by magnetic-loss absorption in the presence of Fe or Ni catalyst particles and dielectric absorption in pinewood/moisture, enabling rapid, volumetric heating.^41^ Microwaves cause polar molecules (such as water and hydroxyl groups in cellulose) to rotate rapidly, generating heat internally. This can lead to higher temperatures in localized areas of the biomass, enabling the decomposition of cellulose, hemicellulose, and lignin more efficiently than conventional heating. Water in pinewood absorbs microwave radiation and quickly heats up, causing rapid evaporation and decomposition of the biomass due to its high dielectric constant. This can reduce the drying time significantly compared with conventional thermally heated gasification. As the biomass is heated under microwave radiation, it undergoes pyrolysis, where the cellulose, hemicellulose, and lignin are cracked into gases and char. All pyrolysis and volatilization reactions take place until 250 °C. Thereafter, the remaining solid is known as char. The rapid heat-transfer rate associated with microwave energy means that devolatilization happens more rapidly and smaller volatile compounds may be formed at lower temperatures than conventional heating methods. Cellulose and hemicellulose decomposition are similar to thermal gasification; microwave heating induces the breakdown of cellulose into levoglucosan, furans, and other gases. However, microwave operation promotes catalytic tar cracking/reforming, converting condensables into smaller, lighter molecules more effectively than conventional heating.^41–43^ The decomposition of lignin in microwave gasification reactions (eqs 9–11) occurs between 275 to 550 °C and follows a similar pattern to thermal gasification, but the increased rate of heating may promote more efficient breakdown of larger aromatic hydrocarbons, potentially reducing tar formation. During microwave gasification, the gasification reactions, eqs 9–11 (partial oxidation and water gas reactions), take place in a manner similar to thermal gasification but with more rapid heating and at potentially lower operating temperatures. Microwave heating can more efficiently promote partial oxidation and the water gas shift reaction, leading to higher yields of CO and H_2_. These reactions produce syngas more efficiently under microwave conditions, particularly because of the uniform and localized heating. Microwave gasification typically results in lower tar formation (eqs 12–16) compared with conventional thermal gasification. Rapid heating leads to a more efficient breakdown of tar compounds into syngas (eqs 12–16), with less accumulation of complex organic molecules. As with thermal gasification, higher temperatures and longer reaction times promote the cracking of larger tar molecules into smaller gases. The rapid heating in microwave gasification leads to less tar accumulation and cleaner syngas output.

### 3.2 Gas, Char, and Tar Yields in Non-catalytic and Catalytic Gasification

The gas, char, and tar yields of gasification are shown in Figure 3. As shown in Figure 3a, the gas, char, and tar yields in non-catalytic microwave gasification are 82.41%, 13.3%, and 4.29%, respectively. The gas yield includes both syngas and other light gases. Microwave energy causes rapid internal heating and vaporization, leading to faster pyrolysis and gas formation. The non-catalytic thermal-assisted gasification, as shown in Figure 3b, results in a gas yield of 78.169%, a char yield of 14.299%, and a tar yield of 7.5309%, respectively. Thermal gasification relies on conductive heat transfer, which is slower than microwave heating.

**Figure 3:**
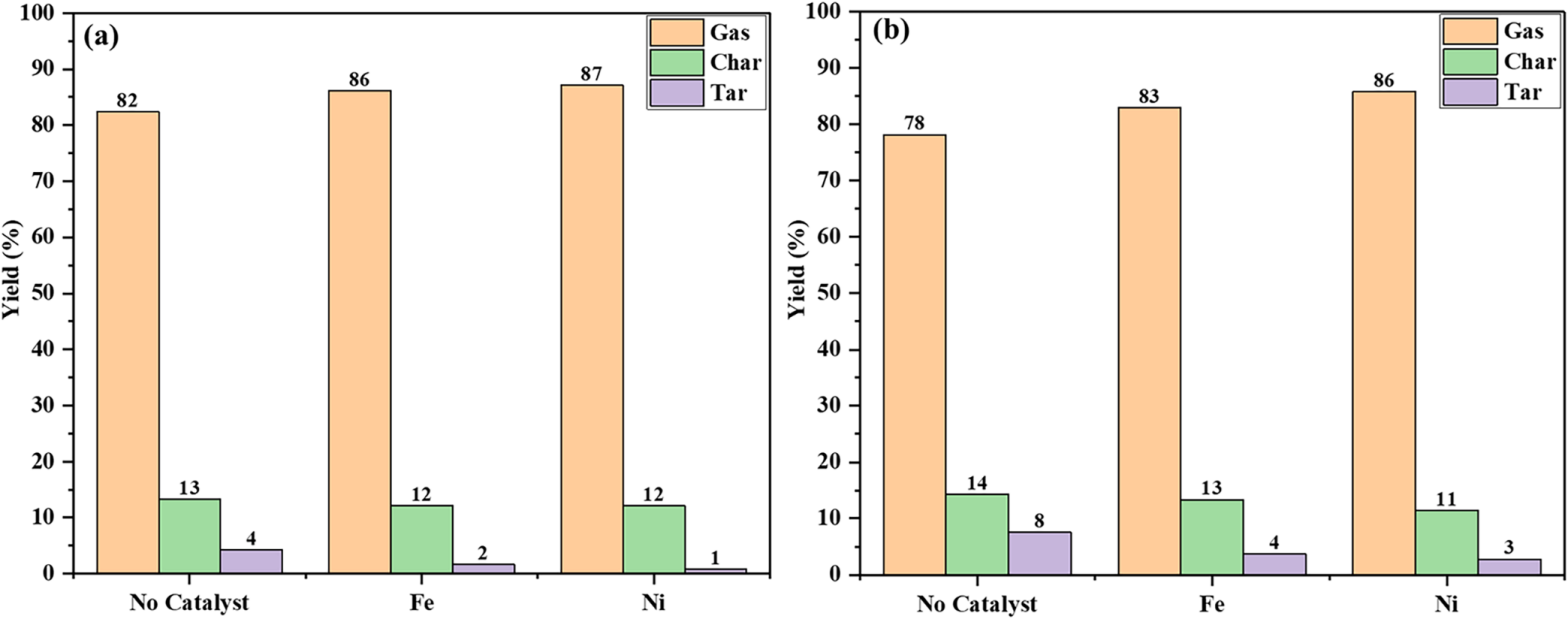
Gas, char, and tar yields of pinewood gasification: (a) microwave and (b) conventional gasification.

The catalytic microwave-assisted gasification using the synthesized iron catalyst is shown in Figure 3a. The gas, char, and tar yields were 86.151%, 12.2%, and 1.649%, respectively. In contrast, thermally assisted pinewood gasification with the iron-based catalyst, as shown in Figure 3b, yielded 82.936% gas, 13.346% char, and 3.7178% tar. For catalytic microwave-assisted gasification with the synthesized nickel catalyst, the gas yield was 87.107%, char yield was 12.1%, and tar yield was less than 1% (0.793%). In comparison, thermal-assisted gasification with a nickel catalyst resulted in a gas yield of 85.796%, char yield of 11.43847%, and tar yield of 2.76454%. Microwave gasification can produce more tar in the initial stages due to rapid heating, which leads to the formation of volatile organic compounds and heavy aromatic compounds. At higher temperatures, these compounds are fully decomposed into gases by microwave radiation. This process may promote the formation of complex tar molecules, especially when the temperature is not uniformly distributed or when the thermal degradation of intermediate products occurs faster than they can be fully converted into gas. In contrast, thermal gasification, with its slower heat transfer, allows more time for decomposition into lighter gases, resulting in lower tar production.

### 3.3 Gas Production in Non-catalytic Gasification of Pinewood

In this work, we used pinewood as a biomass, and this pinewood comes from pine forests, which is a sustainable and renewable resource contributing to carbon sequestration as part of the natural carbon cycle. The carbon released during the gasification of pinewood can be balanced by the carbon absorbed by pine trees during their growth. This makes pinewood gasification a relatively sustainable option, contributing less to net atmospheric CO_2_ compared to fossil fuels. Additionally, forest thinning and pinewood waste management can help improve forest health while providing a valuable source of biomass for gasification. Pinewood has a relatively high energy density compared with other types of biomass. Its carbon content is typically higher and has a good balance of volatile matter and fixed carbon. Pinewood also contains a significant amount of lignin, a complex polymer that is more resistant to degradation, meaning it tends to produce a higher yield of syngas when gasified. This is beneficial for generating high-quality gas that can be used for power generation or chemical synthesis. Furthermore, pinewood has a low ash content compared to other biomass types, such as agricultural residues or grass. The lower ash content reduces the risk of slagging and fouling in the gasifier, making the gasification process more efficient and less prone to equipment issues.^40^

Figure 4a–d shows the non-catalytic lignocellulosic pinewood gasification under microwave heating. As the heating of pinewood begins, we observe that, at 160 °C, 22.153% CO and 76.647% CO_2_ are formed due to combustion reactions (eqs 2 and 3). As the temperature increases, we observe that CO_2_ decreases and CO increases, indicating that reversible Boudouard reactions and hydrogenation reactions (eqs 9 and 10) occur up to 275 °C. After reaching 300 °C, we observe that the gasification reaction begins and the water gas shift and steam methane reforming reactions take place, continuing up to 350 °C. After 350 °C, no further changes are observed during biomass gasification, and we find that the gas composition is stabilized with 2.092% hydrogen, 29.469% methane, 32.236% carbon monoxide, and 25.369% carbon dioxide. Beyond 350 °C, the gasification reaction does not continue because pinewood does not have a sufficiently high dielectric constant. Pinewood’s dielectric constant is between 3 and 4, which is relatively low compared to the requirements for microwave-driven reactions, thus necessitating catalytic gasification.

**Figure 4:**
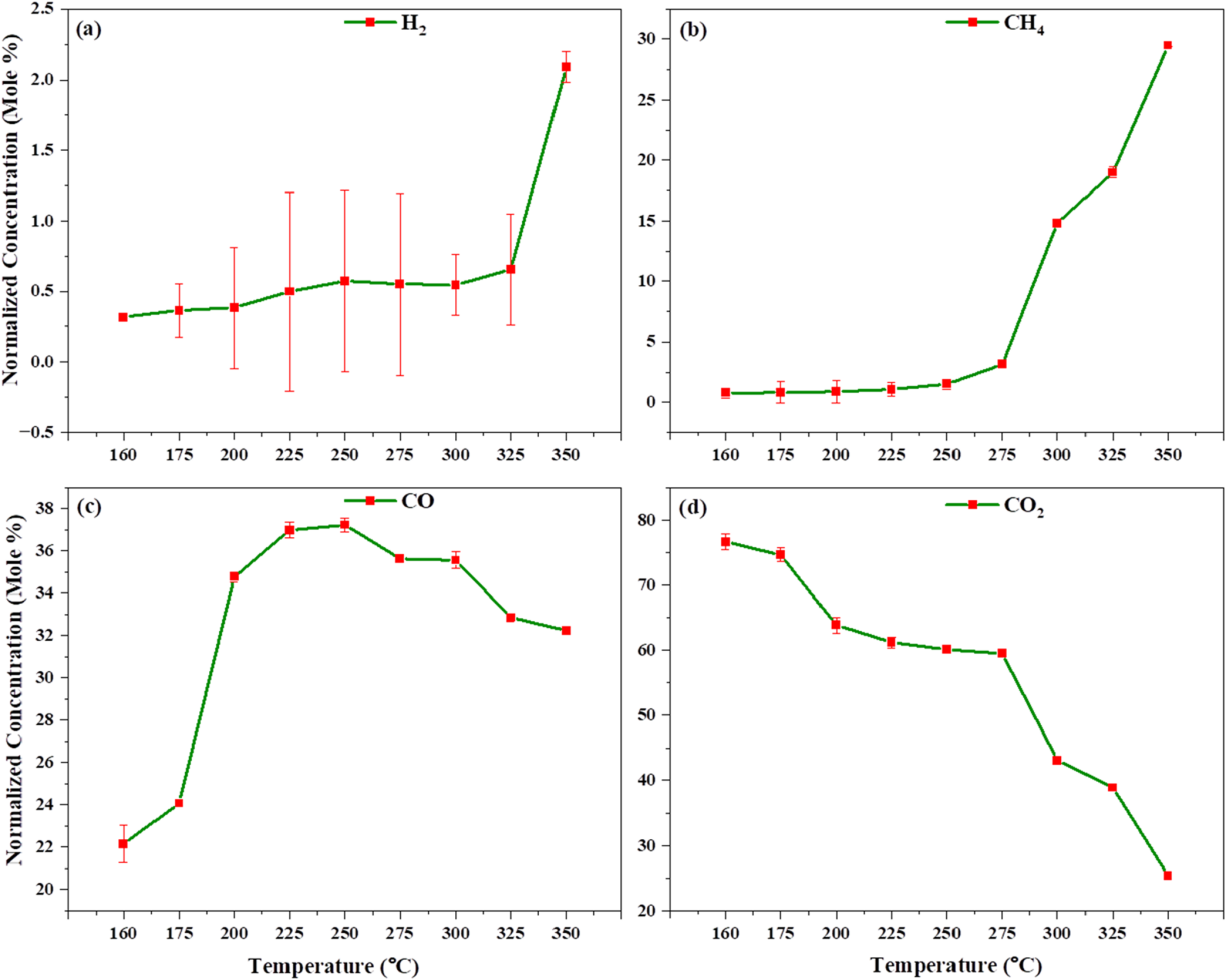
Effect of temperature on non-catalytic biomass gasification of pinewood by microwave-driven heating: (a) H_2_, (b) CH_4_, (c) CO, and (d) CO_2_ normalized concentration vs. temperature.

Figure 5a–d shows the non-catalytic gasification of thermally assisted heating. Initially, at 360 °C, we observe that the concentration of CO is 33.197% and that of CO_2_ is 55.145%, indicating that combustion reactions (eqs 2 and 3) occur up to 360 °C. As the temperature increases, we observe that hydrogenation and heterogeneous water–gas reactions take place, producing hydrogen and methane. The absence of an external gasifying agent requires additional energy for the dissociation of C–C, C=C, and C–H bonds, resulting in higher temperatures being required for gasification without a catalyst. Gasification without a catalyst, using the internal oxygen content present in pinewood, allows the water–gas reaction to occur, but at a very low extent. At 570 °C, we observe a decrease in carbon monoxide and carbon dioxide concentrations, while hydrogen and methane concentrations increase due to the water gas shift reaction. At this stage, gasification reactions also begin. After 600 °C, char gasification reactions (eqs 9, 10, and 11) occur, including hydrogenation, reverse Boudouard reaction, and heterogeneous water–gas reactions. At higher temperatures, tar cracking reactions also take place, where higher-molecular-weight molecules break down into smaller molecules like syngas and other lighter molecules. Consequently, the gas composition stabilizes at 900 °C with 36.674% hydrogen, 9.966% methane, 27.452% carbon monoxide, and 24.018% carbon dioxide, respectively.

**Figure 5:**
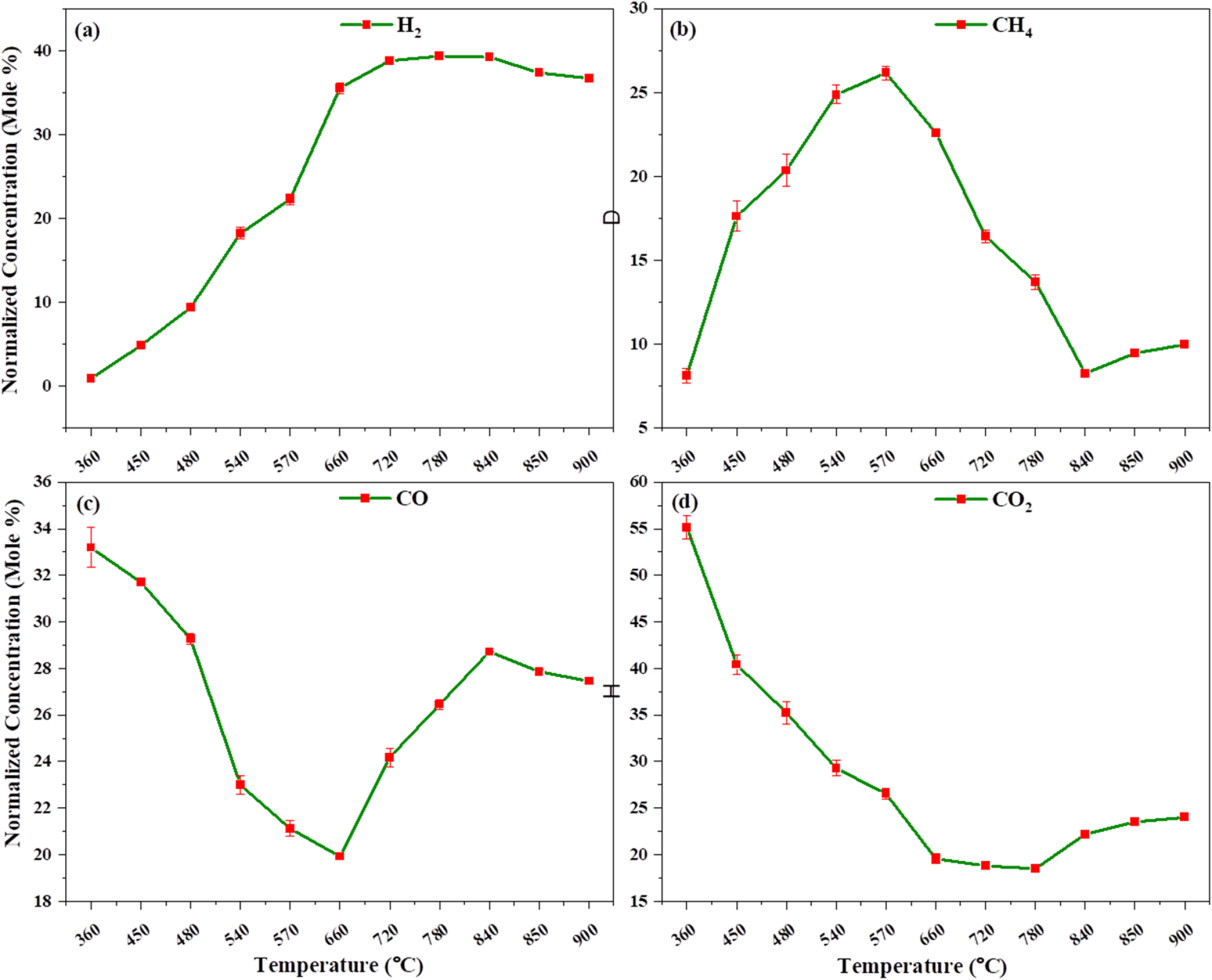
Effect of temperature on non-catalytic gasification in thermal-assisted heating: (a) H_2_, (b) CH_4_, (c) CO, and (d) CO_2_ normalized concentration vs. temperature.

### 3.4 Gas Production in Catalytic Gasification of Pinewood

The selection of a catalyst is crucial for pinewood gasification. We have reviewed a significant amount of literature and found several catalysts mentioned, including iron, nickel, molybdenum, cobalt, palladium, platinum, and others. Based on previous studies,^6,14,44–47^ iron- and nickel-based catalysts were chosen because these two type of catalysts are particularly effective in promoting the water gas shift and Boudouard reactions, which produce more syngas and facilitate tar cracking reactions. Both catalysts are effective in dry reforming and tar cracking, and they also have good thermal stability. Iron and nickel catalysts are widely used in the synthesis of syngas from biomass gasification, as they facilitate key chemical processes, namely, the water gas shift reaction, the reversible Boudouard reaction, and the steam reforming of hydrocarbons. Both iron and nickel catalysts have a dielectric constant of nearly one at 2450 MHz, which is characteristic of conductive materials. The dielectric constant measures a material’s ability to store electrical energy in an electric field. In metals, electrons are free to move, which allows them to conduct electricity very well. Thus, the dielectric constant of iron and nickel is essentially one, indicating that they are good conductors of electricity at higher frequencies. At these frequencies, metals such as iron and nickel also exhibit magnetic properties that influence their permeability rather than their dielectric constant. Nickel, in particular, has the highest magnetic permeability. Both iron and nickel catalysts are very effective in microwave applications due to their magnetic properties, which allow them to interact with microwaves and produce localized heating through a magnetic-loss mechanism.

#### 3.4.1 Performance of the Iron Catalyst

Figure 6a–d shows the catalytic gasification of lignocellulosic pinewood using an iron catalyst. We observe that under microwave heating at 160 °C, partial oxidation of carbon and complete oxidation reactions of biomass occur. At this temperature, 35.186% carbon monoxide (CO), 32.561% carbon dioxide (CO_2_), 26.035% hydrogen (H_2_), and 4.827% methane (CH_4_) are obtained. This indicates that combustion reactions (eqs 2 and 3), the water gas shift reaction, and gasification reactions take place at 160 °C. As the temperature increases, CO and CO_2_ decrease, while H_2_ increases; CH_4_ shows a slight decline. This is primarily due to the water gas shift reaction and the Boudouard reaction, which continue until 275 °C. At 275 °C, we find the highest hydrogen concentration. However, some additional reactions, such as decarbonization, occur which slightly increases the methane concentration. The reverse Boudouard reaction also takes place, leading to an increase in carbon monoxide and a decrease in hydrogen due to the reversible water gas shift reaction. At 275 °C, we observe the highest hydrogen concentration, but the pinewood is not completely gasified and a significant amount of char remains. Therefore, we increased the temperature and found that at 525 °C the biomass is gasified completely. At this temperature, the gas composition stabilizes with 46.693% hydrogen, 32.932% carbon monoxide, 3.145% methane, and 16.358% carbon dioxide. When an iron catalyst was used, we observed that the methane concentration remained below 5%, indicating that methanation reactions do not occur as significantly as in thermal heating.

**Figure 6:**
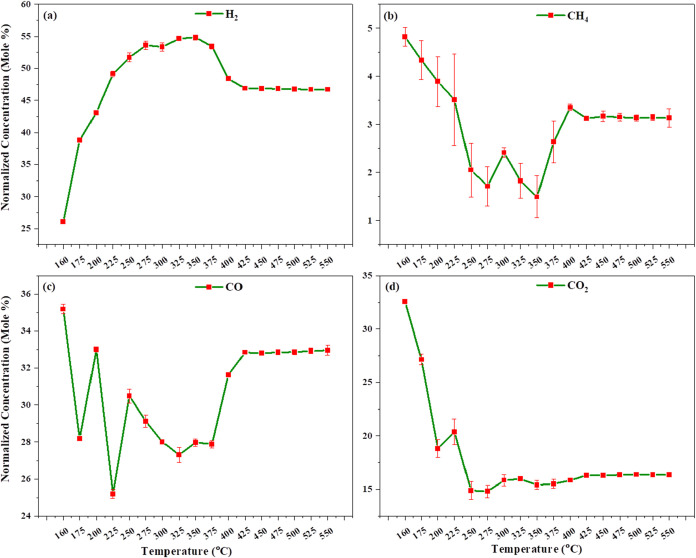
Effect of temperature on catalytic gasification using an Fe catalyst in microwave heating: (a) H_2_, (b) CH_4_, (c) CO, and (d) CO_2_ normalized concentration vs. temperature.

Figure 7a–d illustrates the gasification of lignocellulosic biomass with an iron catalyst at atmospheric pressure. When iron is used as a catalyst with pinewood, it lowers the activation energy of the reaction. As shown in Figure 7a–d, at 350 °C, the concentrations of CO_2_ and CO are higher compared to gasification with pinewood alone. Methane and hydrogen start forming at 350 °C, and as the temperature increases to 570 °C, hydrogen production increases significantly. At this point, equilibrium is achieved for the water gas shift (WGS) reaction. Subsequently, a tar cracking reaction occurs, leading to more CO_2_ formation. This reaction is reversible, causing hydrogen to be converted back into CO, resulting in a decrease in hydrogen concentration after reaching 720 °C. At higher temperatures, hydrogen concentration increases due to tar conversion reactions that occur at higher temperatures when using the iron catalyst. Additionally, some char reacts with hydrogen to form methane after 800 °C, as seen in eq 9. The dry reforming reaction is partially reversible, leading to a decrease in the CO concentration and an increase in the CO_2_ concentration. After reaching 900 °C, the syngas composition stabilizes with 42.261% H_2_, 7.5167% CH_4_, 39.952% CO, and 9.6562 mol % CO_2_.

**Figure 7:**
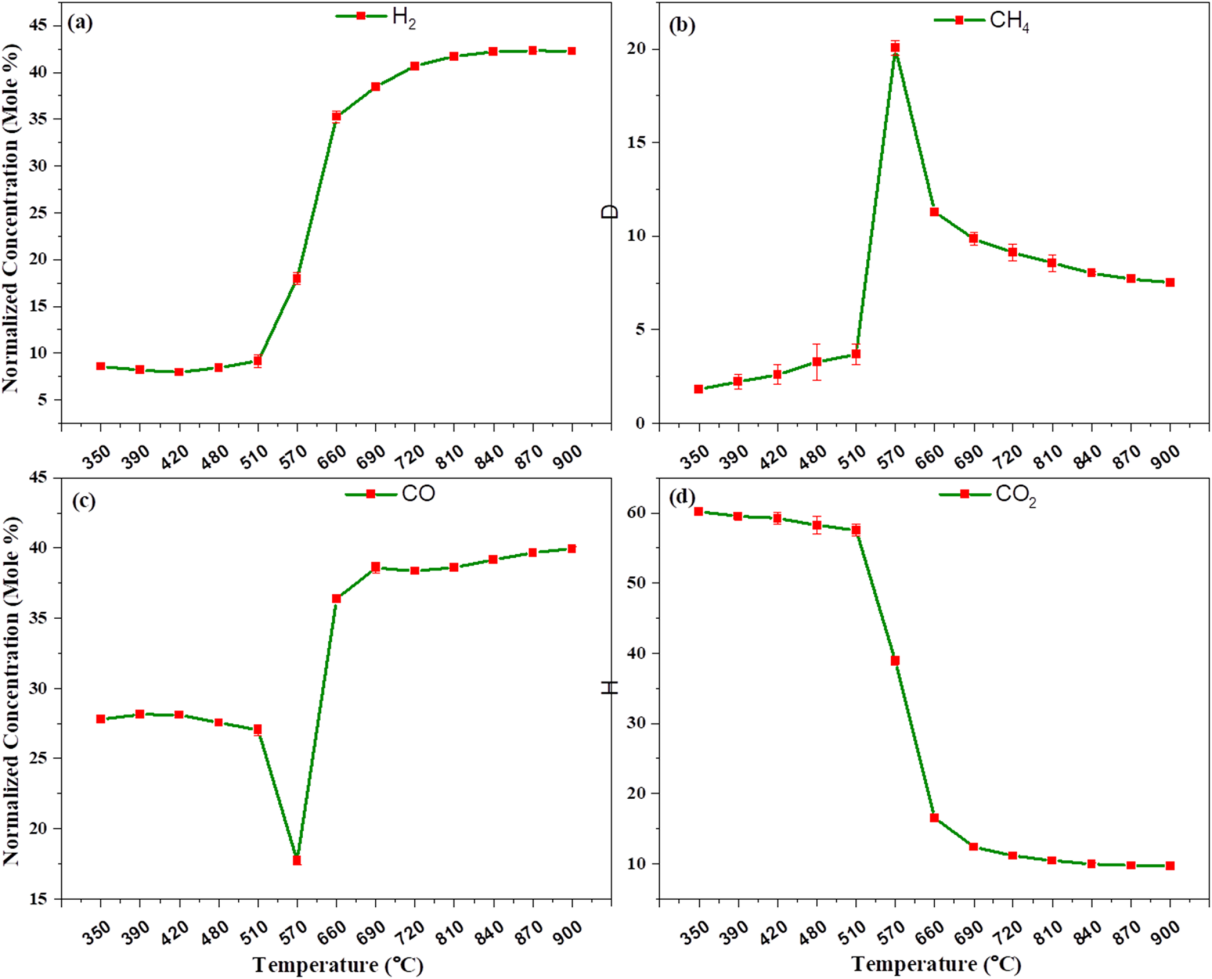
Effect of temperature on catalytic gasification using an iron catalyst in conventional heating: (a) H_2_, (b) CH_4_, (c) CO, and (d) CO_2_ normalized concentration vs. temperature.

#### 3.4.2 Performance of the Nickel Catalyst

Figure 8a–d shows the catalytic gasification of lignocellulosic pinewood using a nickel catalyst. The nickel catalyst is also highly effective for tar cracking and enhances syngas production through the water gas shift reaction. Additionally, it serves as an excellent catalyst for steam methane reforming. Upon initially heating the lignocellulose pinewood, we observe partial oxidation of carbon and complete combustion of carbon occurring at 160 °C. At this temperature, we detect 45.704% carbon monoxide, 20.032% carbon dioxide, 26.779% hydrogen, and 4.376% methane. As the temperature increases, we find that up to 275 °C carbon dioxide decreases, while hydrogen and methane concentrations increase, reaching 42.652% hydrogen and 4.668% methane. Afterward, as the temperature continues to rise, carbon monoxide and hydrogen concentrations increase, while methane and carbon dioxide decrease due to the ongoing Boudouard reaction and the water gas shift reaction. At 525 °C, the syngas composition observed is 53.964% H_2_, 2.366% CH_4_, 38.507% CO, and 4.661% CO_2_. Beyond 525 °C, continued water gas shift and Boudouard reactions shift the composition slightly; at 550 °C, the gas composition is 40.583% H_2_, 5.484% CH_4_, 33.895% CO, and 18.237 mol % CO_2_.

**Figure 8:**
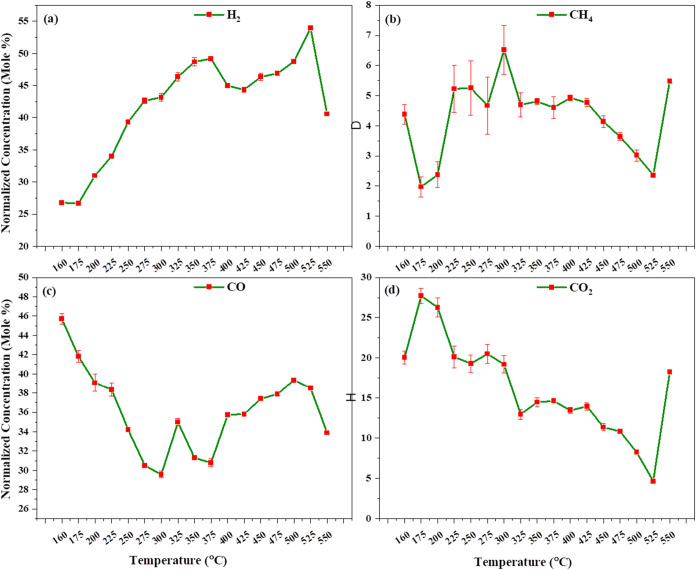
Effect of temperature on catalytic gasification using a nickel catalyst in microwave-driven heating: (a) H_2_, (b) CH_4_, (c) CO, and (d) CO_2_ normalized concentration vs. temperature.

Figure 9a–d illustrates the gasification of lignocellulosic biomass with a nickel catalyst at atmospheric pressure, Initially, as seen in Figure 9a–d, CO_2_ and CO reach their peak concentrations around 360 °C. As the temperature increases, the concentrations of CO_2_ and CO decrease due to the water gas shift reaction (eq 3) and char gasification reactions (eqs 9–11), which subsequently increases the concentrations of hydrogen (H_2_) and CO. At 900 °C, we observe that combustion reactions are more pronounced compared to that at lower temperatures. This is because the catalyst lowers the activation energy, promoting the conversion of CO_2_ and CO into hydrogen. As a result, hydrogen concentrations increase significantly but then slightly decrease due to the water gas shift reaction (eq 3). Meanwhile, the CO concentrations decrease initially but later increase again, while the methane (CH_4_) concentration decreases due to steam methane reforming, converting methane into CO and H_2_. At 880 °C, the highest hydrogen concentration (54.986%) is observed using the nickel catalyst. Finally, at 900 °C, the syngas composition is 49.518% H_2_, 7.147% CH_4_, 27.108% CO, and 15.043% CO_2_. Also, the effect of temperature at various Fe and Ni catalyst loadings in both systems is presented in Figures S2–S5 (Table 2).

**Figure 9:**
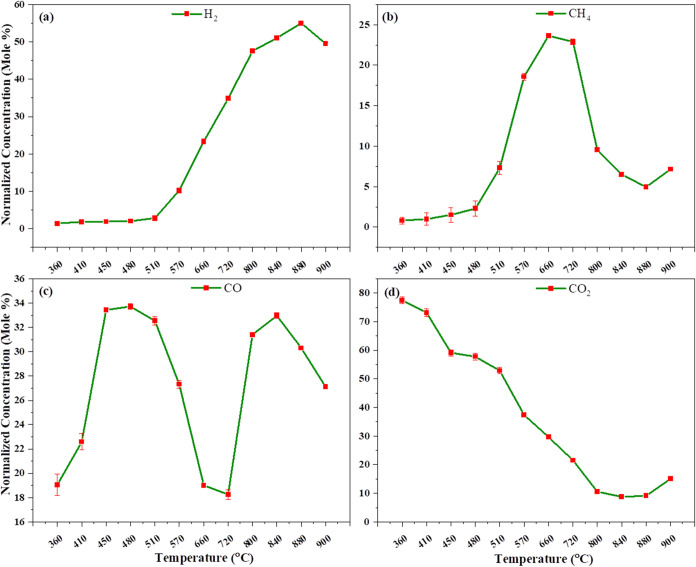
Effect of temperature on catalytic gasification using a nickel catalyst in conventional heating: (a) H_2_, (b) CH_4_, (c) CO, and (d) CO_2_ normalized concentration vs. temperature.

**Table 2:** Comparison of Syngas Concentration from Woody Biomass Gasification in Conventional and Microwave Heating

catalyst	heating method	temperature (°C)	H_2_ (%)	CO (%)	CH_4_ (%)	CO_2_ (%)	ref
no catalyst	conventional	750	28.72	40.22	15.49	15.56	^30^
Fe–Ni/AC	conventional	750	46.46	23.64	9.15	20.74	^30^
Fe_2_O_3_/charcoal	conventional	700	39.00	31.00	9.00	22.00	^31^
Co–Ni/MHC	microwave	500	37.92	48.8	5.87	7.34	^43^
Fe–Co/MHC	microwave	500	28.67	52.44	8.39	10.48	^43^
Fe–Ni/MHC	microwave	500	29.45	50.99	8.00	4.63	^43^
Fe	conventional	900	42.26	39.95	7.52	9.66	this study
Ni	conventional	900	49.52	27.11	7.15	15.04	this study
Fe	microwave	550	46.69	32.96	3.13	16.34	this study
Ni	microwave	525	53.96	38.51	2.37	4.66	this study

### 3.5 X-ray Diffraction

Figure 10 illustrates the X-ray diffraction results of pinewood and catalysts (Fe and Ni). Figure 10a–c shows the XRD patterns of pinewood, char remaining in pinewood using conventional heating, and char remaining in pinewood using microwave heating, respectively. As shown in Figure 10a, the XRD pattern of pinewood typically shows distinct peaks corresponding to the crystallographic planes of cellulose (identified by JCPDS#: 00-061-2828). It typically shows two prominent peaks corresponding to the (101) and (002) crystallographic planes at 2θ angles of 12.9° and 22.8°, respectively. Another peak at a 2θ angle of 42.61° (identified by JCPDS#: 00-061-2828) with a crystal plane of (100) shows the weak graphitic properties of carbon. Figure 10b,c shows peaks of char remaining after gasification in microwave and conventional heating, respectively. Similarly, Figure 10a,b shows similar peaks of pinewood but with reduced intensities. Also, in the conventional heating sample, a sharp graphitic peak is found at 42.61°, indicating its more crystalline nature. Figure 10d presents the XRD pattern of the synthesized nickel catalyst; it shows peaks corresponding to Ni (identified by JCPDS#: 00-001-1258), indicating its cubic structure; the peaks are detected at 2θ angles of 44.68°, 52.08°, 76.5°, 93.32°, and 98.5°, and these peaks correspond to the crystal planes with Miller indices of (1 1 1), (2 0 0), (2 2 0), (3 1 1), and (2 2 2), respectively. Also, in Figure 10f, similar peaks are found in microwave heating with little noise in the baseline due to char. Thus, we observe that Ni is not converted into nickel oxides. Also, no carbon peak is observed around 22.8° and 43.2°. In Figure 10e, we can observe that both peaks of Ni (identified by JCPDS#: 00-001-1258) and NiO_2_ (identified by JCPDS#: 01-085-1977) are found during thermal heating. Ni is detected from peaks at 2θ angles of 44.68° and 98.6°, and these peaks correspond to the crystal planes with Miller indices of (1 1 1) and (2 2 2), respectively. NiO_2_ is detected from peaks at 2θ angles of 44. 08°, 65.08°, 82.34°, and 82.61°, and these peaks correspond to the crystal planes with Miller indices of (1 0 4), (1 1 0), (0 2 4), and (0 1 11), respectively. In Figure 10g is presented the XRD pattern of the synthesized iron catalyst; the peaks corresponding to Fe (identified by JCPDS#: 00-001-1262) indicate that it has a cubic structure; the peaks are detected at 2θ angles of 44.89°, 65.21°, 82.46°, and 99.09°, which correspond to the crystal planes with Miller indices of (1 1 0), (2 0 0), (2 11), and (2 2 0), respectively. In both heating modes, Fe peaks (identified by JCPDS#: 00-001-1262) remain; weaker Fe_2_O_3_ reflections (identified by JCPDS#: 00-024-0072) are also observed. All peaks of Fe are detected, and Fe_2_O_3_ peaks are also found at 2θ angles of 43.47°, 49.41°, and 82.46°; these peaks correspond to the crystal planes with Miller indices of (2 0 2), (024), and (0 2 10), respectively. Also, one more peak is observed around 22.8°, which is due to coke or char. Raised background consistent with amorphous carbon is observed.

**Figure 10:**
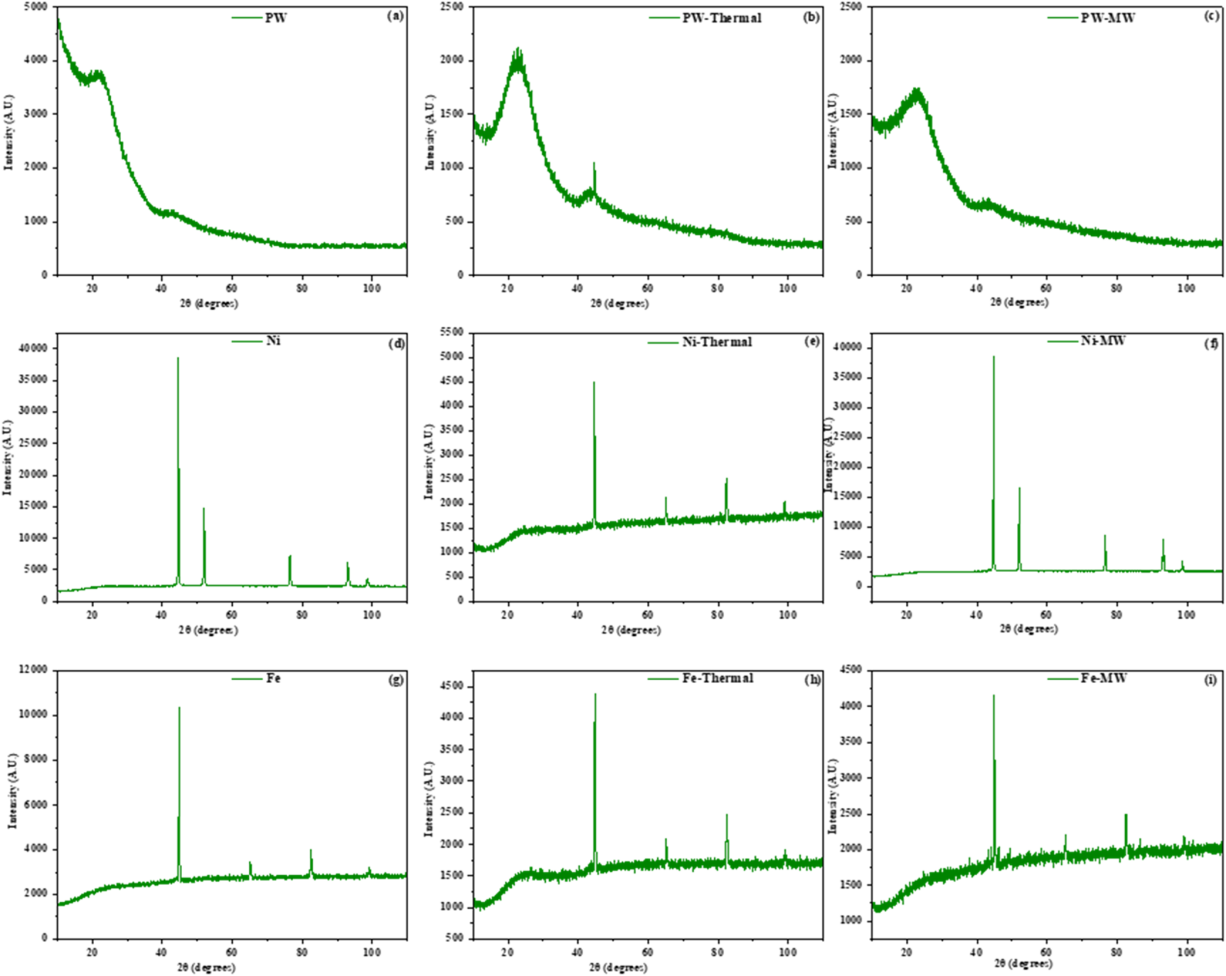
XRD patterns of (a) PW, (b) PW-thermal heating, (c) PW-MW heating, (d) Ni catalyst, (e) Ni-thermal heating, (f) Ni-MW heating, (g) Fe catalyst, (h) Fe-thermal heating, and (i) Fe- MW heating.

## 4 Conclusion

The performance of gasification of pinewood under both microwave and conventional heating methods was compared. Pinewood gasification was carried out in both heating systems with and without a catalyst. The study explored the decomposition of pinewood during gasification in microwave-driven and conventional heating separately to better understand the reactivity of pinewood with or without a catalyst during gasification. We compared the quality of syngas and cold gas efficiency in microwave and conventional heating; Tables S1 and S2 clarify how pinewood forms and consumes char under oxygen-lean conditions without external oxidants. In microwave gasification, pinewood produced higher syngas (54 mol % H_2_, 39 mol % CO, 2 mol % CH_4_, and 5 mol % CO_2_ using a Ni catalyst), whereas conventional heating produced syngas (50 mol % H_2_, 27 mol % CO, 7 mol % CH_4_, and 15 mol % CO_2_ using a Ni catalyst) with more CO_2_ and CH_4_. Microwave gasification resulted in a higher gas yield and lower tar yield than conventional gasification. Under microwave heating, localized hot spots over Ni efficiently drive reforming and water gas shift reactions at ∼525 °C, yielding H_2_-rich syngas; Fe assists via oxygen shuttling but is not the primary source of high H_2_. Microwave gasification improved the reactivity, increased the gas yield, increased the energy recovery efficiency, and reduced the tar yield compared to conventional methods. Microwave heating achieved an energy recovery efficiency of 68.31%, while for conventional heating it was 52.68% using a Ni catalyst. The percentage increase in energy recovery efficiency with microwave heating was 29.66%. These advantages are attributed to selective heating and the formation of hot spots within the catalytic gasification of pinewood, accelerating reaction rates without raising the overall temperature. The microwave-assisted process is more efficient for small-scale, modular applications as traditional gasifiers rely on economies of scale for commercial viability. A microwave gasifier offers significant advantages for modular, on-demand, syngas production.

## Supplementary Material



## Notes

Energy recovery efficiency comparison in microwave vs conventional heating and the effect of feed-to-catalyst ratio (PDF)
