# Supplementary material for: Sustainable H2‑Rich Syngas Production via Microwave-Assisted vs Conventional Catalysis of Pinewood
Source: Ind Eng Chem Res. 2026 Jan 5;65(2):1059–73. doi: 10.1021/acs.iecr.5c02616 (PMC12828724; doi:10.1021/acs.iecr.5c02616)
Supplement: Supplementary file 1 [file ie5c02616_si_001.pdf]

Supplementary Information

Sustainable H_2-Rich Syngas Production via Microwave-Assisted vs. Conventional Catalysis of Pinewood

Kshitij Tewari[1], Devin Burton[2], Brandon Robinson[1], Changle Jiang[1], Debangsu Bhattacharyya[1], Jianli Hu[1]*

[1]Department of Chemical and Biomedical Engineering, West Virginia University, Morgantown, WV 26505, United States

[2]Department of Physics and Astronomy, West Virginia University, Morgantown, WV 26505, United States

*Corresponding Author: john.hu@mail.wvu.edu

For Energy Input Calculations:

Ramp time to setpoint:

- Conventional: $\Delta T = 900 - 25 = 875$ °C → time = 87.5 min (1.458 h)
- Microwave: $\Delta T = 550 - 25 = 525$ °C → time = 52.5 min (0.875 h)

Energy to reach setpoint (ramp only):

Conventional:

$E_{wall} = P_{wall} \times t \approx 1.120$ kW×1.458 h = 1.633 kWh = 5.88 MJ.

Microwave :

- Delivered reflected power (best case, if reflection ≈ 0): $E_{delivered, MW} \approx 0.30$ kW × 0.875 h = 0.2625 kWh = 0.945 MJ
- If ~10% reflected: $E_{delivered, MW} \approx 0.851$ MJ
- Wall-plug (depends on generator efficiency; typical 55-70%): $E_{wall,MW} \approx 1.35$–1.72 MJ

We added a quantitative input energy analysis. Based on our 10 °C min^{-1} ramps, the conventional furnace requires ~5.88 MJ (wall-plug, upper bound from nameplate) to reach 900 °C, while the microwave requires 0.85-0.95 MJ delivered reflected power (depending on reflection) and ~1.35-1.72 MJ wall-plug to reach 550 °C.

Energy recovery efficiency in Microwave vs. Conventional heating

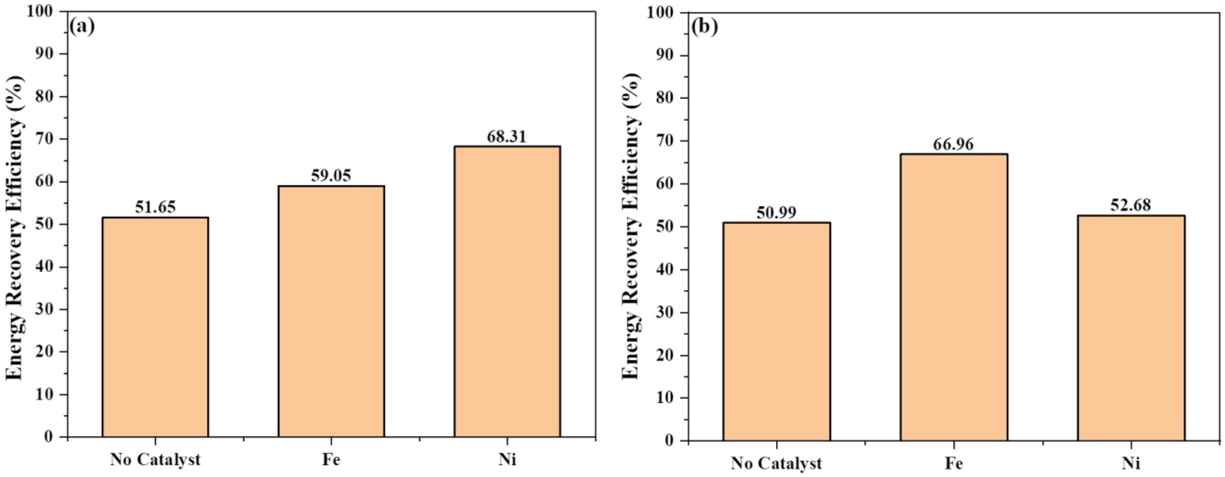

Figure S1. Energy recovery efficiency (a) Microwave (b) Conventional

Figure S1 shows energy recovery efficiency in Microwave vs. Conventional heating.

Gas Production in catalytic gasification of Pinewood: Effect of Feed to catalyst ratio

1. Effect of Fe Catalyst in Microwave heating

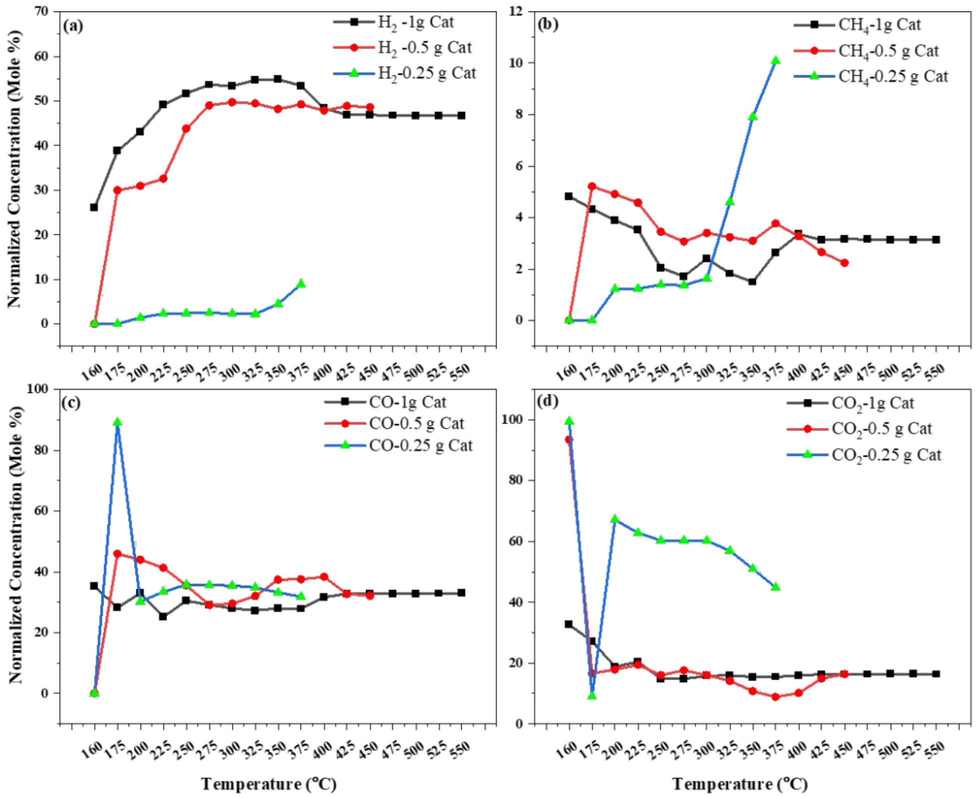

Figure S2. Effect of Feed to Fe catalyst ratio using Microwave heating

2. Effect of Ni Catalyst in Microwave heating

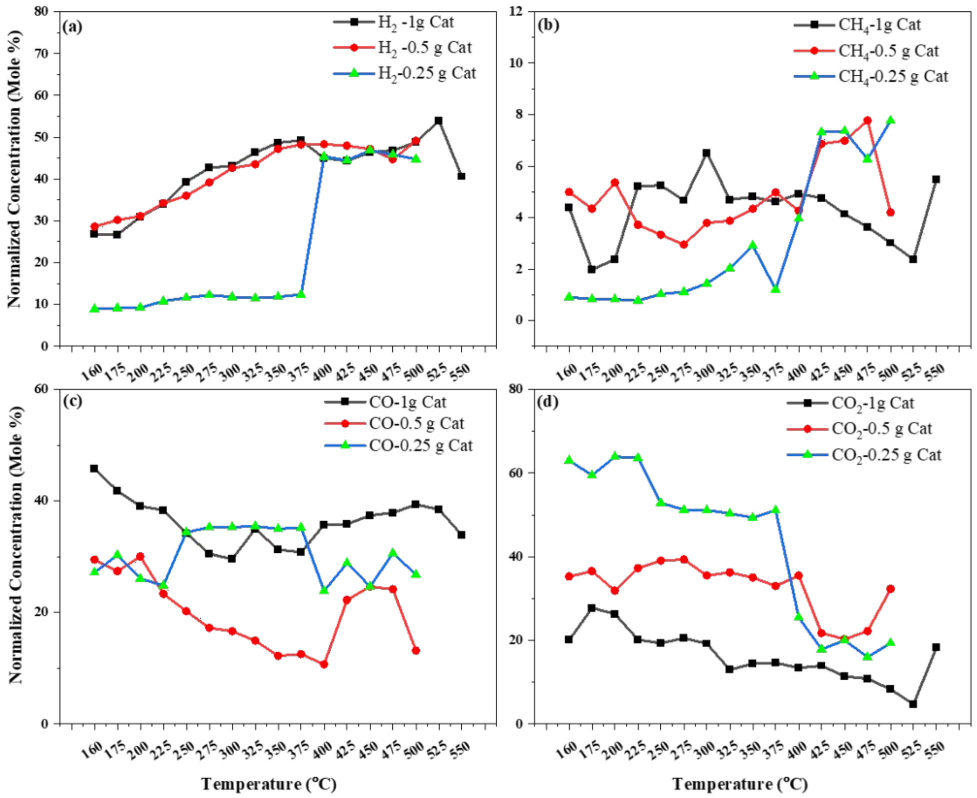

Figure S3. Effect of Feed to Ni catalyst ratio using Microwave heating

3. Effect of Fe Catalyst in conventional heating

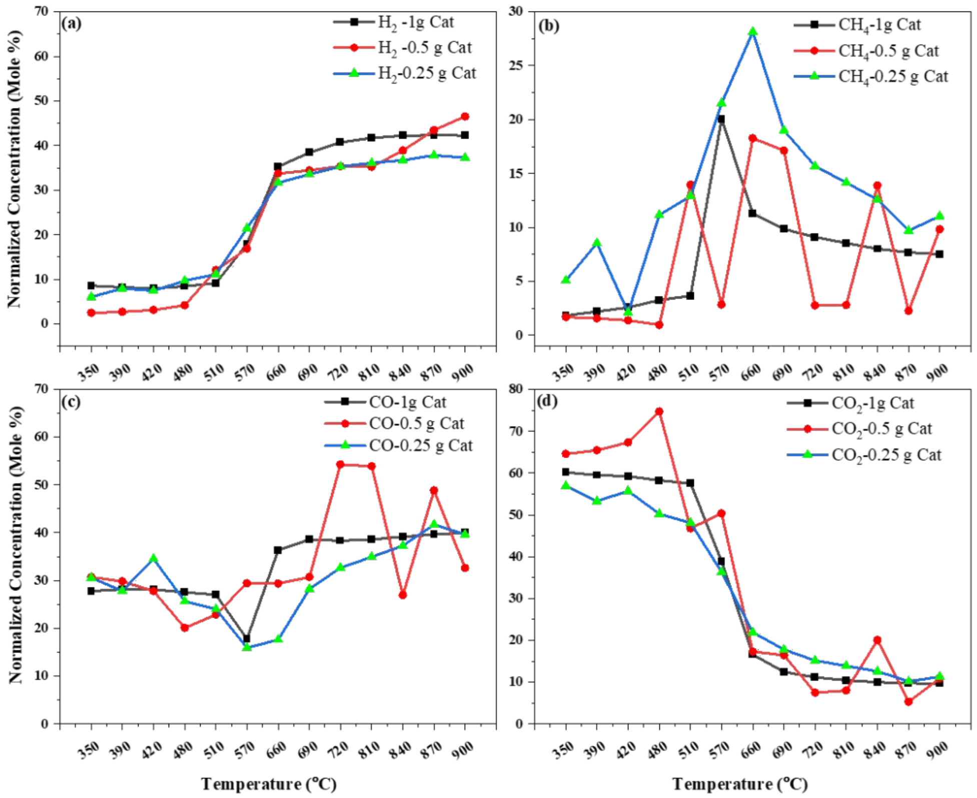

Figure S4. Effect of Feed to Fe catalyst ratio using conventional heating

4. Effect of Ni Catalyst in Conventional heating

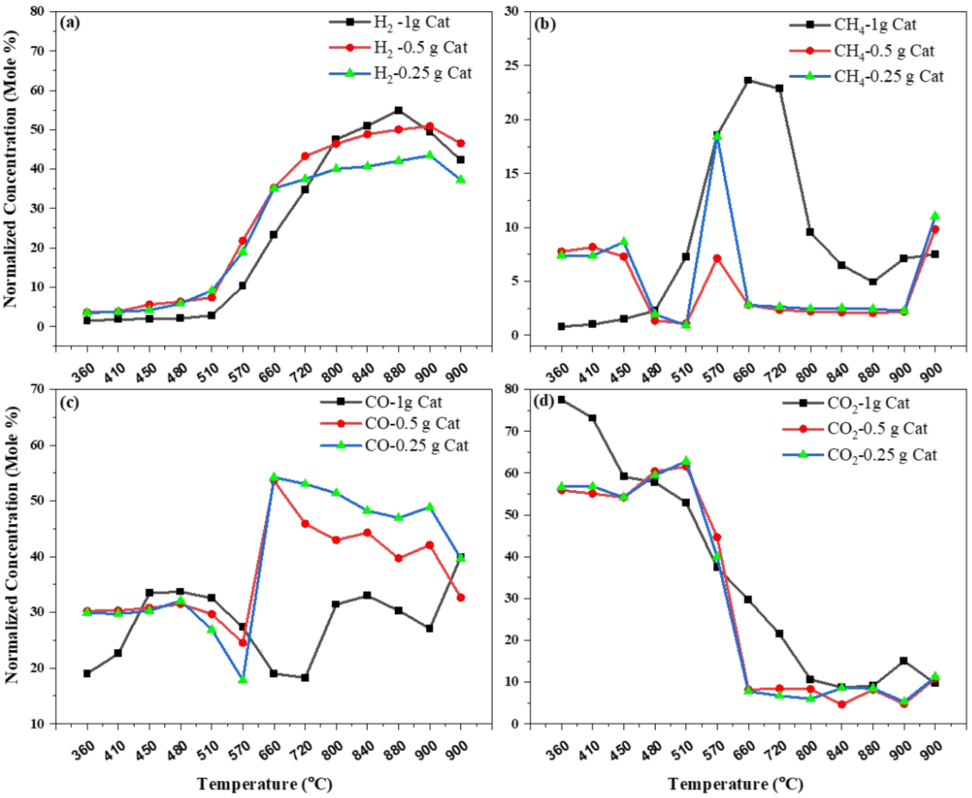

Figure S5. Effect of Feed to Ni catalyst ratio using conventional heating

The feed-to-catalyst ratio plays a crucial role in determining the efficiency of pinewood gasification under both microwave and conventional heating modes. The comparative results for Fe and Ni catalysts under varying catalyst loadings (0.25 g, 0.5 g and 1.0 g) highlight important differences in heating mechanisms and catalytic behavior.

In the case of microwave heating **(**Figures S2 and S3), catalyst loading exerts a strong influence on overall reaction performance. At lower catalyst amounts (0.25 g), insufficient microwave absorption occurs, since metallic particles are the primary microwave absorbers that convert electromagnetic radiation into localized thermal energy. Particularly for Fe catalyst, the low dielectric loss and less magnetic loss at small quantities results in incomplete reactor heating, slower reaction initiation, and limited syngas production. As the catalyst amount is increased, more localized “hot spots” are formed, which enhances pyrolysis and gasification of

the biomass. This effect becomes most pronounced at 1.0 g catalyst loading, where rapid internal heating accelerates devolatilization and tar cracking, leading to a sharp increase in H_2 and CO concentrations while simultaneously suppressing CH_4 and CO_2 formation. For Ni catalyst, this dependence is even stronger, as Ni not only improves microwave coupling but also actively catalyzes steam reforming and the water-gas shift reaction. At high catalyst loadings, Ni promotes deep tar cracking and methane reforming, yielding a H_2-rich syngas with lower levels of hydrocarbons and CO_2. These results emphasize that under microwave-driven gasification, catalyst loading is directly impacts the heating efficiency of the system, with 1 g catalyst identified as the optimal loading for stable conversion in the designed reactor**.**

By contrast, under conventional heating (Figures S4 and S5), the role of catalyst loading is less significant. Since heating is externally driven by the furnace via conductive and convective heat transfer, the bulk reactor temperature is primarily controlled by the set furnace conditions rather than by microwave absorption. Consequently, increasing catalyst loading from 0.25 g to 1.0 g produces only marginal changes in syngas composition. Both Fe and Ni catalysts exhibit relatively constant hydrogen and CO levels across the tested loadings, indicating that once a minimum surface area is available for tar cracking and reforming reactions, additional catalyst mass does not substantially enhance conversion. This trend highlights the fundamentally different role of catalyst loading in conventional heating, where the catalyst acts only as a chemical promoter rather than as a microwave absorber and localized heat source.

A comparison between Fe and Ni catalysts, Fe catalyst under microwave heating demonstrates improved syngas yield with increasing loading, but the dependence is more moderate than Ni due to Fe's weaker catalytic activity for methane reforming and water-gas shift reactions. Nevertheless, higher Fe loading ensures better heat distribution, supporting partial oxidation and Boudouard reactions that sustain syngas formation. Ni catalyst, on the other hand, exhibits superior performance in microwave systems, particularly at 1 g loading, where its dual function as a microwave absorber and strong reforming catalyst generates the most H_2-rich syngas across all studied conditions. In conventional heating, Ni still outperforms Fe in terms of H_2 yield, but the effect of catalyst loading remains minimal, consistent with the heat-transfer-dominated process.

Overall, these observations demonstrate that the feed-to-catalyst ratio is a critical parameter in microwave-assisted gasification, where sufficient catalyst loading ensures effective microwave absorption, rapid heating, and enhanced syngas production. In contrast, conventional heating is far less sensitive to catalyst mass, since heat transfer occurs externally, and catalyst primarily governs chemical pathways rather than reactor heating efficiency.

Also, a comparative study of syngas quality with Fe and Ni catalysts under microwave and conventional heating below in Table S1.

Table S1: Comparative Syngas Quality

**Catalyst**	**Heating Method**	**Temp (°C)**	**H_2 (mol%)**	**CO (mol%)**	**CH_4 (mol%)**	**CO_2 (mol%)**	**H_2/CO Ratio**	**Notes on Syngas Quality**
Fe	Microwave	550	46.69	32.96	3.13	16.34	1.42	Moderate H_2, low CH_4; decent H_2/CO ratio for FT/methanol
Ni	Microwave	525	53.96	38.51	2.37	4.66	1.4	Highest H_2 and CO, very low CH_4/CO_2 - best syngas quality

Fe	Conventional	900	42.26	39.95	7.52	9.66	1.06	Balanced H_2-CO but high CH_4, lower H_2/CO ratio
Ni	Conventional	900	49.52	27.11	7.15	15.04	1.83	High H_2, but also high CO_2/CH_4; good H_2/CO for WGS or H_2 recovery

Table S2: Calculation of cold gas efficiency for conventional heating Vs Microwave heating

**Case**	**y_H2**	**y_CO**	**y_CH4**	**y_CO2**	**Biomass LHV (MJ/kg)**	**LHV_syngas (MJ/Nm3)**	**CGE (%)**
MW–Ni (525 °C)	0.540	0.385	0.024	0.047	14.960	11.528	69.355
Conv–Ni (900 °C)	0.495	0.271	0.072	0.150	14.960	11.324	68.124
MW–Fe (550 °C)	0.467	0.330	0.031	0.163	14.960	10.316	62.062
Conv–	0.423	0.400	0.075	0.097	14.960	12.290	73.937

Fe (900 °C)							
